# IntraCoronary Artery Retrograde Thrombolysis vs. Thrombus Aspiration in ST-Segment Elevation Myocardial Infarction: Study Protocol for a Randomized Controlled Trial

**DOI:** 10.3389/fcvm.2022.928695

**Published:** 2022-09-15

**Authors:** Mingzhi Shen, Jihang Wang, Dongyun Li, Xinger Zhou, Yuting Guo, Wei Zhang, Yi Guo, Jian Wang, Jie Liu, Guang Zhao, Shihao Zhao, Jinwen Tian

**Affiliations:** ^1^Department of Cardiology, Hainan Hospital of Chinese People’s Liberation Army (PLA) General Hospital, Hainan Geriatric Disease Clinical Medical Research Center, Hainan Branch of China Geriatric Disease Clinical Research Center, Sanya, China; ^2^The Second School of Clinical Medicine, Southern Medical University, Guangzhou, China; ^3^The First Department of Health Care, Second Medical Center, PLA General Hospital, Beijing, China; ^4^Department of Cardiology, Second Medical Center, PLA General Hospital, Beijing, China; ^5^Department of Critical Medicine, Hainan Hospital of Chinese PLA General Hospital, Sanya, China; ^6^Tongji Medical College, Huazhong University of Science and Technology, Wuhan, China

**Keywords:** ST-segment elevation myocardial infarction, intracoronary artery retrograde thrombolysis, thrombus aspiration, reperfusion preconditioning, percutaneous coronary intervention (PCI)

## Abstract

**Background:**

Type 2 diabetes (T2DM) is a major risk factor for myocardial infarction. Thrombus aspiration was considered a good way to deal with coronary thrombus in the treatment of acute myocardial infarction. However, recent studies have found that routine thrombus aspiration is not beneficial. This study is designed to investigate whether intracoronary artery retrograde thrombolysis (ICART) is more effective than thrombus aspiration or percutaneous transluminal coronary angioplasty (PTCA) in improving myocardial perfusion in patients with ST-segment elevation myocardial infarction (STEMI) undergoing primary percutaneous coronary intervention (PPCI).

**Methods/Design:**

IntraCoronary Artery Retrograde Thrombolysis (ICART) vs. thrombus aspiration or PTCA in STEMI trial is a single-center, prospective, randomized open-label trial with blinded evaluation of endpoints. A total of 286 patients with STEMI undergoing PPCI are randomly assigned to two groups: ICART and thrombus aspiration or PTCA. The primary endpoint is the incidence of >70% ST-segment elevation resolution. Secondary outcomes include distal embolization, myocardial blush grade, thrombolysis in myocardial infarction (TIMI) flow grade, and in-hospital bleeding.

**Discussion:**

The ICART trial is the first randomized clinical trial (RCT) to date to verify the effect of ICART vs. thrombus aspiration or PTCA on myocardial perfusion in patients with STEMI undergoing PPCI.

**Clinical Trial Registration:**

[https://www.chictr.org.cn/], identifier [ChiCTR1900023849].

## Background

Patients with type 2 diabetes (T2DM) are at high risk and have a poor prognosis of myocardial infarction, especially after ST-segment elevation myocardial infarction (STEMI). STEMI is due to plaque rupture leading to intracoronary thrombosis, thus blocking the coronary artery (1, 2). The strategy to treat myocardial infarction is to open the infarct-related coronary artery to achieve reperfusion as soon as possible (3). However, in the process of opening the occluded vessels, it leads to myocardial damage. This phenomenon is called reperfusion injury (4). Myocardial death caused by reperfusion accounts for 25% of the total death area. Animal experiments show that the proportion is even as high as 50% (5). Reperfusion injury can cause reperfusion arrhythmia, myocardial stunning, microvascular occlusion, intramyocardial hemorrhage, and lethal myocardial reperfusion injury (6).

At present, methods of opening occluded vessels include intravenous thrombolysis, primary percutaneous coronary intervention (PPCI), and emergency coronary artery bypass grafting (CABG) (7, 8). The advantage of intravenous thrombolysis is that it can be implemented in general hospitals and even primary hospitals, and can be carried out quickly before hospitalization or even in ambulances (9–11). However, the success rate of intravenous thrombolysis is relatively low (12, 13). Even after intravenous thrombolysis success, emergency percutaneous coronary intervention (PCI) should be performed within 24 h, which increases the risk of hemorrhagic events or even fatal hemorrhage. Therefore, intravenous thrombolysis was gradually reduced or even stopped in the hospitals that could perform primary PCI in time or quickly transfer to primary PCI hospitals (7). Because of the complexity of CABG and the high requirement of patients’ own conditions, it is relatively difficult to carry out emergency CABG (8).

Primary percutaneous coronary intervention is still the best way to treat acute myocardial infarction because of its simple operation and exact effect (14). Thrombus load is an independent risk factor for PPCI. Slow-flow and no-reflow after stent implantation affect the prognosis (15). There are two ways to deal with thrombus before PPCI. The first is thrombus aspiration (16). However, routine thrombus aspiration before PPCI does not reduce the rate of all-cause mortality, rehospitalization of myocardial infarction, or stent thrombosis, and even increase the risk of stroke (17). Even in patients with high thrombus burden, routine thrombus aspiration does not improve outcomes (18). The second is to give antithrombotic or thrombolytic agents through the transcatheter, such as glycoprotein (GP) IIb/IIIa inhibitors, alteplase, et al. (19). However, due to the blood flow erosion, antithrombotic agents, and thrombolytic agents cannot stay for a long time at the thrombus site, affecting the drug effect; if the dosage is increased, the hemorrhagic risk will increase at the same time (20). It has also been studied that thrombolytic drugs are given after thrombus aspiration (21), the efficacy is similarly affected for the above reasons. There is still a long way to go to solve the problem of intracoronary thrombus, especially in patients with large thrombus burdens.

The sequence of continuous thrombosis is white thrombus, mixed thrombus, and red thrombus. The white thrombus in the head is mainly composed of platelets. The red thrombus at the tail end is mainly composed of red cells within the fibrin network (22). According to the characteristics of thrombus, we put forward the method of ICART, applied it to a small sample population, and achieved a certain effect (23). Only 10% of intravenous thrombolysis is needed for the ICART procedure. Before the newly formed thrombus is destroyed, a very high concentration of thrombolytic agent is produced in the red thrombus of the occluded vessel, which could stay for a long time, resulting in a very high thrombolytic efficiency, and reducing the incidence of no-reflow and slow-flow. At the same time, due to the slow opening of occluded vessels, there is a reperfusion preadaptation, which can reduce the reperfusion injury in theory. At the same time, low-dose thrombolysis does not increase the risk of hemorrhage and stroke. ICART may provide a new plan to solve thrombus burden in STEMI. However, there were only eight cases in the previous study (23). Therefore, we intend to verify the effect of ICART vs. thrombus aspiration or PTCA on myocardial perfusion in patients with STEMI undergoing PPCI.

## Study Design

**FIGURE 1:**
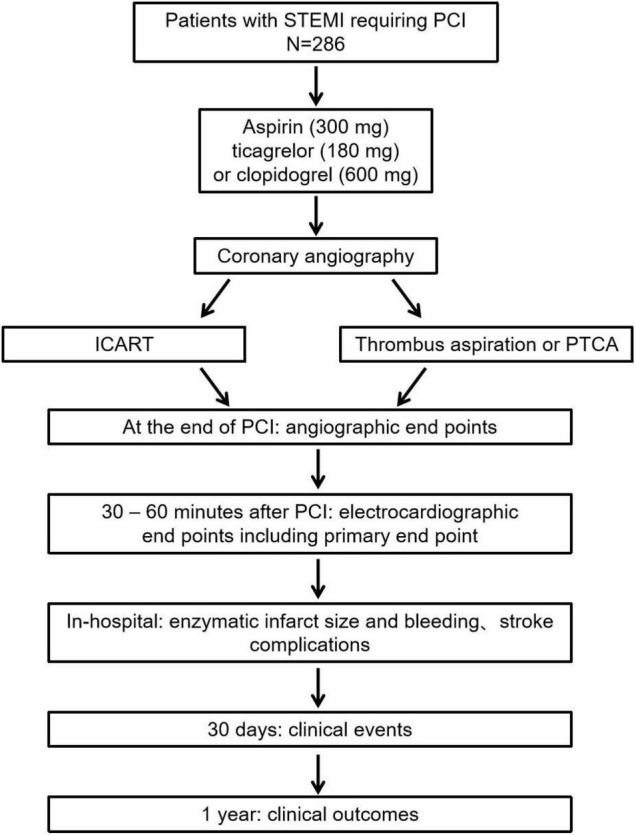
The ICART trial flow chart. ICART, intracoronary artery retrograde thrombolysis; STEMI, ST-segment elevation myocardial infarction; PCI, percutaneous coronary intervention; PTCA, percutaneous transluminal coronary angioplasty.

The ICART trial is a single-center, prospective, randomized controlled trial with a blinded evaluation of endpoints (Figure 1). A total of 286 patients with STEMI undergoing PPCI were randomly divided into ICART or thrombus aspiration or PTCA groups. Randomization is performed by the Empower network random system when deciding to perform PCI. The study was approved by the institutional committee on human research of the Chinese People’s Liberation Army General Hospital and complies with the declaration of Helsinki. The protocol of this trial has been registered at chictr.org.cn (ChiCTR1900023849).

## Study Population

All consecutive STEMI patients who are ready to receive primary PCI are suitable for inclusion. The inclusion criterion includes: (1) STEMI with chest pain lasting 30 min to 12 h; (2) older than 18 years old; (3) volunteered and signed informed consent. The exclusion criteria includes: (1) active peptic ulcer; (2) severe hepatic or renal insufficiency; (3) pregnancy; (4) uncontrolled hypertension; (5) rescue PCI after thrombolytic therapy; (6) unable to sign informed consent; (7) less than 18 years old; (8) left main coronary artery disease; (9) cardiogenic shock; (10) mechanical complications after myocardial infarction (e.g., interventricular septum perforation, papillary muscle rupture); (11) recent history of major surgery, trauma, hemorrhagic disease, cerebrovascular accident, or thrombocytopenia; (12) previous history of CABG; (13) other obvious abnormal signs, laboratory tests, and clinical diseases. According to the judgment of the clinician, the patients are not suitable for the study.

## Preparation of Visualized Thrombolytic Agents and Process of Intracoronary Artery Retrograde Thrombolysis

As we previously described (23), visualized thrombolytic agents are made by dissolving 100,000 units urokinase or 5 mg prourokinase with 15 ml physiological saline and 5 ml iopromide.

**FIGURE 2:**
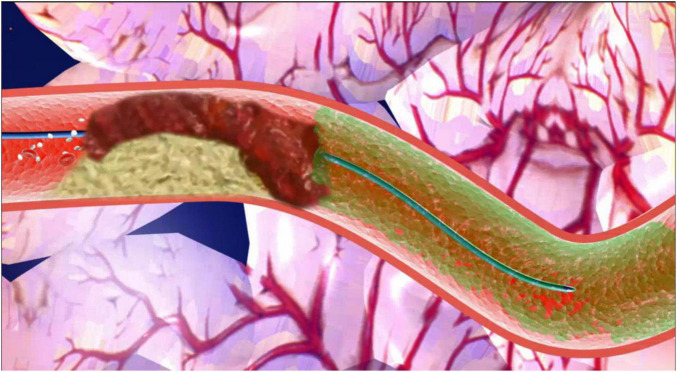
Pattern diagram of intracoronary artery retrograde thrombolysis system. After the passing of the wire, a microcatheter or a cut balloon is sent to the occluded section through the guidewire. One milliliter of thrombolytic cocktail is bolus-injected through the microcatheter to the distal lumen of the vessel, which is repeated every 30 s.

The flow diagram for the ICART system is shown in Figure 2. Coronary angiography identifies the occluded coronary artery. First, the guidewire is sent to the distal end of the culprit’s vessel. A microcatheter or a cut balloon is sent to the occluded section through the guidewire. One milliliter of thrombolytic cocktail is bolus-injected through the microcatheter, which is repeated every 30 s. The effect of thrombolysis is observed by X-ray.

## Treatment

Before PCI, ICART or thrombus aspiration is decided according to the grouping. Thrombus is aspirated by the Export aspiration catheter (Medtronic Inc., Santa Rosa, CA, United States) as previously described (24). Finally, a stent is implanted. In certain patients, pre- or postdilatation with a balloon may be necessary.

Patients are given a loading dose of aspirin (300 mg) and ticagrelor (180 mg) or clopidogrel (600 mg), and are diagnosed STEMI by electrocardiography. It’s up to the operator to decide whether to use heparin or bevaludine for anticoagulation. In the setting of STEMI, radial access is preferred. In patients with radial artery pathways, sheaths are pulled out immediately following the PCI procedure. The femoral approach is reserved for patients without the radial approach. Sheaths are exchanged immediately at the end of the PCI procedure by the Angio-Seal device (St. Jude Medical, Inc., St. Paul, MN, United States). After PCI, tirofiban is used for 36 h. Then low molecular weight heparin is given for 1–3 days after tirofiban. According to the international guidelines, standard post PCI medication includes aspirin (100 mg), ticagrelor (90 mg bid), or clopidogrel (75 mg), beta-blockers, lipid-lowering drugs, and angiotensin converting enzyme inhibitors or angiotensin II receptor blockers (8).

### Electrocardiography

A standard 18-lead ECG is acquired at first medical contact. A standard 12 lead ECG is performed 30–60 min after PPCI. Times of symptom manifestation, arrival at the hospital, guidewire passing, thrombolysis, balloon dilatation, end of PCI, and ECG are recorded. The magnitude of ST-segment deviation is calculated 60 ms from the J-point. The ST segment of post-intervention ECG at 30–60 min is compared with that of the ECG at presentation. ST-segment elevation resolution is defined as complete (>70%), partial (30–70%), or absent (<30%) (Trials 2009, 10:90). Residual ST-segment deviation after PCI is counted as the sum of residual ST-segment depression and elevation in all leads (25). All ECG data are analyzed by a physician who is blinded to the clinical grouping and data.

## Coronary Angiography

The baseline, peri-, and post-procedural angiographic features will be recorded: the presence of thrombus, treatment of a non-culprit vessel during the same procedure, myocardial blush grade (MBG), thrombolysis in myocardial infarction (TIMI) flow grades, side branch occlusion, the presence of angiographically visible distal embolization, minimum lumen diameter, stent diameter, stent length, no-reflow, and slow flow. TIMI flow grades are evaluated according to the previously described method (26). The evaluation of thrombus adopts the previous evaluation standard (27). The thrombus is assessed according to TIMI thrombus classification: 0 = none, 1 = suspected thrombus, 2 = thrombus, linear size ≤ 1/2 vessel diameter, 3 = thrombus, 1/2 ≤ linear size < two times of vessel diameter, 4 = thrombus, linear size ≥ two times of vessel diameter, 5 = complete occlusion due to thrombosis. MBG’s evaluation is based on the previous description (28): 0 = no myocardial staining, 1 = slight myocardial staining, or contrast density, 2 = moderate myocardial staining or contrast density, but less than that of a contra- or ipsilateral non-infarct-related area, and 3 = normal myocardial staining or contrast density, comparable with that of a contra- or ipsilateral non-infarct-related area. Continuous myocardial staining indicates that the contrast agent leaks into the extravascular space, which is defined as grade 0. Distal embolization is defined as filling defects and/or abrupt cutoff of distal vessels in the target lesion (29). The coronary angiograms are analyzed by a physician who does not know the treatment allocation and clinical data.

## Infarct Size

The infarct size is estimated by serial monitoring of cardiac markers such as creatine kinase (CK), creatine kinase-MB (CK-MB), and troponin T. Blood samples are taken at baseline and 3, 6, 9, 12, 18, 24, and 48 h after PCI. Peak value, time to peak release as well as area under the curve are determined.

## Endpoints Assessment

The primary endpoint is to observe the probability of ST-segment resolution > 70% acquired before and 30–60 min after PPCI.

Secondary endpoints include:

1. Angiographic endpoints: TIMI flow grade, distal embolization, no reflow, slow flow, side branch occlusion, myocardial blush grade post-PPCI.
2. Electrocardiographic end points: residual ST-segment deviation 30–60 min after primary PCI.
3. Enzymatic infarct size.
4. Mortality and Major Adverse Cardiac Events: All-cause death, cardiogenic death, cardiogenic shock, reinfarction, revascularization of target vessels, malignant arrhythmia, NYHA class IV heart failure, stent thrombosis, and myocardial infarction rehospitalization are to be registered at 30 days and 1 year.

Safety endpoints consist of in-hospital hemorrhagic and stroke complications.

Furthermore, the primary and secondary endpoints will be analyzed in a preset subgroup, which is defined as:

1. Age (<65 vs. >65 years)
2. Gender
3. Smoker
4. Diabetes mellitus
5. ST-segment resolution
6. Number of diseased vessels (multi-vessel vs. single vessel)
7. Proximal lesions
8. Infarct-related artery [left anterior descending artery (LAD) vs. non-LAD]
9. Bivalirudin
10. Ischemic time (<3 vs. >3 h)
11. Initial TIMI thrombus grade
12. Angiographic presence of thrombus
13. Intra-aortic artery balloon pump
14. Left ventricular ejection fraction
15. Type of P2Y12 inhibitor
16. Killip class
17. Symptom onset
18. Pre-procedural TIMI flow
19. Post-procedural TIMI flow
20. Post-PCI myocardial blush grade
21. Pacing use.

## Clinical Follow-Up

All-cause death, cardiogenic death, cardiogenic shock, reinfarction, revascularization of target vessels, stroke, malignant arrhythmia, NYHA class IV heart failure, bleeding complications, stent thrombosis, and myocardial infarction rehospitalization are to be registered at 30 days and 1 year. The follow-up information will be obtained from hospital records as well as by telephone follow-up with the patients and/or their relatives.

## Data Collection and Management

Data and instrumental measurements will be collected from all subjects using an electronic data capture system (EmpowerEDC, Shanghai, China). Data entry and management will be completed by an independent data administrator to guarantee data accuracy. Only the principal investigator could access the data. The final dataset can be acquired only by the principal investigator and the independent statistician. All procedures will comply with the confidentiality standards for medical data. All documents related to clinical trial implementation will be retained by the principal investigator. Important scheme modifications during this project will be communicated to the institutional review board, trial registry, investigators, trial participants, and the journal of publication. Management and analysis of data will be carried out by an independent expert statistician.

## Statistical Considerations

### Sample Size Estimation

It has been reported that the incidence of ST-segment elevation resolution >70% has been reported to be 56.6% in STEMI patients treated with thrombus aspiration (24). We hypothesize that ICART administration during PCI increases the incidence of ST-segment elevation resolution >70–75%. To detect this difference between ICART and thrombus aspiration or PTCA groups, 286 patients are required to reach a 5% significance level (two-sided) with 90% power, which allows 5% of cases to fall off.

### Statistical Analysis

Statistical analysis will be carried out using the SPSS statistical software package (version 18.0; SPSS, Inc., Chicago, IL, United States), and the level of significant difference will be established at α = 0.05. The data analysis will be performed by an independent professional statistician who is blinded to allocation. Analysis of efficacy will include all participants who complete the whole study.

Descriptive statistics will be used to compare the baseline characters between ICART and thrombus aspiration or PTCA group. If the normality test is satisfied, the independent *t*-test will be used; otherwise, the Mann-Whitney U test will be carried out. Regarding the primary and secondary endpoint measures, multiple logistic regression will be used to compare the differences. The mean and standard deviation values of these parameters will be reported.

## Discussion

The ICART project is a single-center, prospective, randomized controlled trial to make clear whether ICART procedure is more effective than thrombus aspiration or PTCA in improving myocardial perfusion and reducing reperfusion injury in STEMI patients undergoing primary PCI. This is the first RCT trial to clarify the effect of ICART vs. thrombus aspiration or PTCA on myocardial perfusion in STEMI patients undergoing PPCI.

## Trial Status

The trial is currently in the recruitment phase. The current protocol version number is V1.0 from 14 June 2019. The start recruitment date is January 2022. The expected end date is December 2023.

## Data Availability Statement

The original contributions presented in the study are included in the article/supplementary material, further inquiries can be directed to the corresponding authors.

## Ethics Statement

This study has been approved by the Ethics Committee Board of the Chinese PLA General Hospital, Beijing, China on 25 March 2014. Written, informed consent to participate will be obtained from all participants.

## Author Contributions

MS designed this project in collaboration with JT, SZ, JW, and DL. MS also drafted this manuscript. DL, XZ, YG, WZ, YG, JW, JL, and GZ revised the manuscript critically. DL was also responsible for the study of some cases in another center. All authors read and approved the final manuscript.

## Conflict of Interest

The authors declare that the research was conducted in the absence of any commercial or financial relationships that could be construed as a potential conflict of interest.

## Publisher’s Note

All claims expressed in this article are solely those of the authors and do not necessarily represent those of their affiliated organizations, or those of the publisher, the editors and the reviewers. Any product that may be evaluated in this article, or claim that may be made by its manufacturer, is not guaranteed or endorsed by the publisher.
